# Optimizing Release of Nucleic Acids of *African Swine Fever Virus* and *Influenza A Virus* from FTA Cards

**DOI:** 10.3390/ijms222312915

**Published:** 2021-11-29

**Authors:** Ahmed Elnagar, Timm C. Harder, Sandra Blome, Martin Beer, Bernd Hoffmann

**Affiliations:** Institute of Diagnostic Virology, Friedrich-Loeffler-Institut, 17493 Greifswald, Germany; Ahmed.Elnagar@fli.de (A.E.); Timm.Harder@fli.de (T.C.H.); Sandra.Blome@fli.de (S.B.); Martin.Beer@fli.de (M.B.)

**Keywords:** *African swine fever virus*, *Influenza A virus*, nucleic acid release, DNA/RNA isolation, direct PCR amplification, FTA cards

## Abstract

FTA cards and related products simplify the collection, transport, and transient storage of biological sample fluids. Here, we have compared the yield and quality of DNA and RNA released from seven different FTA cards using seven releasing/extraction methods with eleven experimental eluates. For the validation, dilution series of *African swine fever virus* (ASFV) positive EDTA blood and *Influenza A virus* (IAV) positive allantoic fluid were used. Based on our data, we conclude that direct PCR amplification without the need for additional nucleic acid extraction and purification could be suitable and more convenient for ASFV DNA release from FTA cards. In contrast, IAV RNA loads can be amplified from FTA card punches if a standard extraction procedure including a lysis step is applied. These differences between the amplifiable viral DNA and RNA after releasing and extraction are not influenced by the type of commercial FTA card or the eleven different nucleic acid releasing procedures used for the comparative analyses. In general, different commercial FTA cards were successfully used for the storage and recovery of the ASFV and IAV genetic material suitable for PCR. Nevertheless, the usage of optimized nucleic acid releasing protocols could improve the recovery of the viral genome of both viruses. Here, the application of Chelex^®^ Resin 100 buffer mixed with 1 × Tris EDTA buffer (TE, pH 8.0) or with TED 10 (TE buffer and Dimethylsulfoxid) delivered the best results and can be used as a universal method for releasing viral DNA and RNA from FTA cards.

## 1. Introduction

The Flinders Technology Associates (FTA^®^) Whatman filter paper cards are based on a chemically-treated cellulose membrane, which lyses cells, their nuclei, and organelles from a variety of sources (e.g., blood, saliva, plant tissue). Upon immediate cell lysis, the released nucleic acid is bound within the supporting material, the card fiber. The matrix protects the nucleic acids from damaging agents (e.g., nucleases, oxidative agents, and bacterial growth) which serves to reduce degradation [1]. They are commercially available in a variety of configurations to meet application requirements and custom configurations. FTA cards are impregnated with chaotropic agents that inactivate infectious agents and reduce the biohazard potential of the sample, thereby minimizing risks of exposure to the technical staff during sample processing. This enables the storage of biological material on FTA cards at room temperature for extended periods. No refrigerators or freezers are required, which significantly reduces storage costs [2]. Samples stored on FTA cards can be shipped through regular postal service with no special handling restrictions, making them a very useful tool for the field collection of biological samples [3]. FTA cards are used in the veterinary field as an alternative method for collecting, transporting, and transiently storing samples for molecular diagnostics and have been applied for many viruses, including avian Influenza [4], *Newcastle disease* [5], *porcine reproductive and respiratory syndrome* [6], *infectious bursal disease* [7], *foot and mouth disease* [8], *rabies* [9], and *African swine fever virus* [10]. However, it should be noted that the risk of cross-contamination of samples on FTA cards is higher compared with liquid samples collected in separate tubes. A further disadvantage of the FTA cards is the less efficient nucleic acid extraction based on the reduced recovery of the DNA and RNA from the filter paper matrix [11]. Although the probability of detecting the pathogens on FTA cards is lower than in fresh samples, the cards offer unique advantages for the collection, transportation, and storage of samples. Different procedures for the release of the nucleic acids from the FTA cards were described by the commercial suppliers or in context with specific viruses and cards [12].

In case of animal epidemics, we need safe and stable transport possibilities. It has been shown that swabs, filter papers, and also FTA cards can be helpful; however, the market and the possibilities of reprocessing are large. The objective of this study was to systematically evaluate the efficacy of release of viral DNA (ASFV in EDTA blood) and viral RNA (IAV in allantoic fluid) from seven brands of FTA cards on the market with seven different methods (eleven eluates). Released nucleic acid was measured via direct qPCR amplification as well as by qPCR after DNA/RNA extraction on a standard automated extraction system.

## 2. Results

For the study, four dilutions (10^−1^ to 10^−4^) of ASFV-positive EDTA blood and IAV-positive allantois fluid in the same sample matrix of healthy donors were used as surrogates for DNA and RNA viruses, respectively. The ASFV EDTA blood sample has been diluted with ASFV-negative EDTA blood, and the IAV-positive sample has been diluted with negative allantoid fluid. For the dilutions of ASFV-positive EDTA blood, Ct values of 21.9, 24.1, 27.3, and 30.4 could be ascertained after standard extraction and qPCR. For the IAV dilution series in allantois fluid, Ct values of 17.1, 21.6, 24.5, and 28.4 were defined. In general, all generated raw data of the study are presented in the Supplemental Materials file (Supplementary Tables S1 and S2), including the data of the internal, positive, and negative controls. For better understanding and easier comparison of the generated data, qualitative and quantitative evaluations were performed, and these are summarized in the table and figures of the main text.

### 2.1. Comparison of Nucleic Acid Releasing/Extraction Methods

The comparison was performed with eleven different eluates derived from seven different releasing methods using seven different FTA cards for the releasing of both viral DNA and RNA. All tested methods were analyzed by the direct qPCR amplification and simultaneously by qPCR amplification after nucleic acid extraction on the KingFisher Flex System.

**Figure 1:**
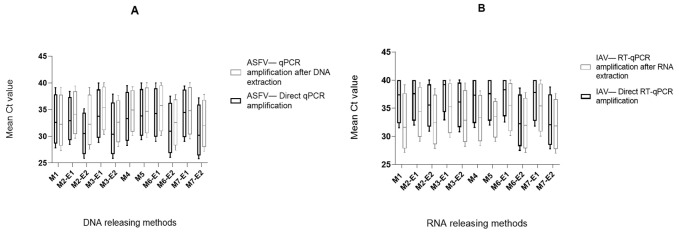
(**A**) Comparison of 11 different releasing methods for the ASFV genome detection with and without nucleic acid extraction. The mean Ct values based on the PCR results of all four dilution steps tested with seven FTA cards are shown. The used methods are described in the legend of Table 1. SD analysis was carried out (number of replicates = 11, Supplementary Table S1) are shown. The standard deviation (SD) value for all methods by the direct qPCR amplification is 1.96 and for qPCR amplification with extraction is 1.72. The standard error of the mean value for all methods by direct amplification is 0.59 and for the amplification with extraction is 0.51. An unpaired multiple *t*-test was performed to test the significance between the different RNA releasing methods based on the both qPCR amplification direct and with extraction with a resulting *p*-value > 0.99 for the taken values, which is not statistically significant. (**B**) Comparison of 11 different releasing methods for the IAV genome detection with and without nucleic acid extraction. The mean Ct values based on RNA-releasing methods of all four dilution steps. SD analysis was carried out (number of replicates = 11, Supplementary Table S1) are shown. Standard deviation (SD) value for all methods by the direct RT-qPCR amplification is 2.43 and for RT-qPCR amplification with extraction is 1.58. The standard error of the mean value for all methods by direct amplification is 0.73 and for the amplification with extraction is 0.47. An unpaired multiple *t*-test was performed to test the significance between the different RNA releasing methods based on the both RT-qPCR amplification direct and with extraction with a resulting *p*-value > 0.99 for the taken values, which is not statistically significant.

The ASFV genome was positively detected via direct qPCR amplification and amplification after DNA extraction by all methods up to the dilution 10^−2^. For the dilutions 10^−3^ and 10^−4^, direct qPCR compared to amplification after additional DNA isolation gave slightly higher numbers of positives (Table 1). As a tendency, the qualitative results were best when using method M2-E2 for direct amplification of the ASFV genome (Table 1). Very similar results could be obtained by the methods M3-E2, M6-E2, and M7-E2, respectively. Thus, all four releasing methods were suitable for the detection of ASFV genome via direct PCR (Table 1 and Supplementary Table S1). The qualitative data (positive vs. negative) were confirmed by the quantitative analysis of the Ct values after direct qPCR and qPCR after extraction. The results of all four dilution steps were combined for the quantitative analyses. Taking qualitative and quantitative data into consideration, direct qPCR outperformed qPCR following DNA extraction (Figure 1A, Supplementary Table S1).

In general, the IAV genome detection via direct RT-qPCR showed more restrictions compared to the amplification results after RNA extraction of the released card eluates. Using the direct RT-qPCR, only the 10^−1^ dilution from all cards gave positive results with all releasing methods. In contrast, the dilution 10^−2^ could only be amplified from all cards if the RNA extraction step for the released eluates was added. The RT-qPCR amplification after RNA extraction demonstrated a higher number of positive results and lower Ct values than the direct amplification, as became clear also for the dilutions 10^−3^ and 10^−4^. The data demonstrated that the methods M6-E2 and M7-E2 had slightly more sensitive results compared to other methods, regarding qualitative and quantitative values (Table 1, Supplementary Table S1 and Figure 1B). Interestingly, these two methods delivered good qualitative and quantitative results also in the direct RT-qPCR. Nevertheless, the positive number of cards and the Ct values were slightly better for the RT-qPCR after extraction compared to the direct RT-qPCR.

To confirm the efficiency and accuracy of the qualitative and quantitative results, a ∆Ct was calculated among the four-dilution series between the values of the released eluates from the FTA cards compared to the same values of the extracted original samples on the KingFisher Flex system, (see Supplementary Table S3). For ASFV, based on the best releasing method, M2-E2 showed tendentially the lowest ∆ Ct value (mean value of the four-dilution series) with a difference of 4.58 after direct qPCR. Two further methods (M3-E2 and M7-E2) showed good results with ∆ Ct value of 4.83 and 4.92, respectively. Good results were defined based on the represented values in the study apart from possession of no statistically significant differences in the results. For IAV-RNA, M6-E2 and M7-E2 demonstrated the most sensitive results for the RT-qPCR amplification of extracted RNA with ∆ Ct differences of 8.96 and 9.15 compared to the original sample fluids (Supplementary Table S3).

### 2.2. Comparison of Different FTA Cards

**Figure 2:**
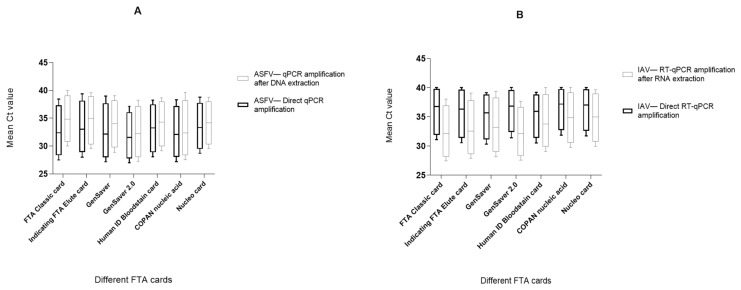
(**A**) Direct qPCR amplification with and without nucleic acid extraction for ASFV detection. The mean Ct values based on different FTA cards. SD analysis was carried out (number of replicates = 7, Supplementary Table S2) are shown. Standard deviation (SD) value for all cards by the direct qPCR amplification is 0.78 and for qPCR amplification with extraction is 1.14. The standard error of the mean value for all methods by direct amplification is 0.29 and for the amplification with extraction is 0.43. An unpaired multiple *t*-test was performed to test the significance between the different FTA cards based on both the qPCR amplification direct and with extraction with a resulting *p*-value > 0.99 for the taken values, which is not statistically significant. (**B**) Direct RT-qPCR amplification with and without nucleic acid extraction for IAV detection. The mean Ct values are based on different FTA cards. SD analysis was carried out (number of replicates = 7, Supplementary Table S2) are shown. Standard deviation (SD) value for all cards by the direct RT-qPCR amplification is 0.76 and for RT-qPCR amplification with extraction is 1.74. The standard error of the mean value for all methods by direct amplification is 0.28 and for the amplification with extraction is 0.55. An unpaired multiple *t*-test was performed to test the significance between the different FTA cards based on the both RT-qPCR amplification direct and with extraction with a resulting *p*-value > 0.99 for the taken values, which is not statistically significant.

In general, all of the FTA cards were comparable to each other and delivered similar results. The ASFV genome could be detected from all card types with the direct qPCR and the qPCR after DNA extraction up to the 10^−2^ dilution. Not all cards spiked with the ASFV dilutions 10^−3^ and 10^−4^ delivered positive amplification results after genome releasing with the 11 methods. This result was independent from the use of direct qPCR or the amplification after additional ASFV-DNA extraction. Nevertheless, the direct qPCR delivered slightly more positive cards and generated the lower Ct values in general (Table 2, Supplementary Table S2 and Figure 2A).

The direct RT-qPCR could detect the IAV genome from all cards using all methods only for the 10^−1^ dilution. However, the RT-qPCR after extraction of the viral RNA was also positive for the dilution step 10^−2^ for all cards and methods. The increased sensitivity of the RT-qPCR including extraction was further supported by analyses of the 10^−3^ and 10^−4^ dilutions. The direct RT-qPCR amplification for IAV showed more negative results and higher Ct values compared to the RT-PCR amplification after RNA isolation (Table 2, Supplementary Table S2 and Figure 2B). The FTA classic card and the GenSaver 2.0 card delivered the best qualitative and quantitative results for the IAV-RNA detection.

Finally, the Ct values defined from the different FTA cards were compared to the Ct values from the extracted original samples on the KingFisher system (Supplementary Table S3). Here, the GenSaver 2.0 card demonstrated the smallest ∆Ct value of 5.87 after the direct qPCR amplification of ASFV-DNA. In addition, the GenSaver 2.0 card and the FTA classic cards delivered the lowest ∆Ct value of 9.43 and 9.48 for RNA amplification after the RNA extraction procedure (Supplementary Table S3).

## 3. Discussion

FTA cards have the advantage of inactivating pathogens and preventing degradation, thus allowing safe transport of the samples and its ability to be mailed as any other document [13]. The feasibility of performing molecular analysis of samples collected on FTA cards has been demonstrated previously [14]. The quality of nucleic acid stored on the cards, the low budget needed for storage and handling, the ease in transporting, and the simple extraction method makes FTA cards a compelling, convenient alternative to traditional methods for the storage and transport of samples [15]. In this study, eleven different methods for the releasing/isolation of DNA/RNA and seven different FTA cards were used and compared for the viral genome detection of ASFV and IAV. There are a variety of purposes for the use of the different FTA cards according to each manufacturer. Some FTA cards are designed for the isolation and purification of nucleic acids, while other cards are consisting of filter papers that are specialized for the collection, transport, and storage of biological samples.

Independent of the different purposes of the cards, our results showed that all types of cards could be used for the isolation of viral DNA and RNA. All tested isolation methods showed comparable yields of DNA via FTA cards for ASFV detection. Among all methods, M2-Eluate 2 (TE buffer + PK + FTA Elute buffer), M3-Eluate 2 (TE buffer + TLR buffer), and M7-Eluate 2 (TED10 + Chelex^®^ 100 Resin) have represented the best values and sensitivity for the detection of ASFV via direct qPCR. Whereas M1 (FTA purification Reagent + PK), M2-Eluate 2 (TE + PK + FTA Elute buffer), M6-Eluate 2, and M7-Eluate 2 showed the best results for IAV detection. Here, the additional extraction of the viral RNA from the released material will improve the qualitative and quantitative detection of the genome. In general, our work correlates with the work of other groups, which used very often TE buffer for the nucleic acid releasing from FTA cards [12,16,17]. Our study showed that the addition of the virotype Tissue Lysis Reagent (TLR) to TE buffer can deliver sensitive results, especially for DNA releasing. The TLR was originally developed for the fast preparation of various sample types without the need for an extraction kit or any complicated nucleic acid isolation procedures [18]. The study of Rodiño et al. (2016) has ascertained the functionality of using FTA purification Reagent for DNA isolation [13]. Surprisingly, this releasing method and the decreases in Ct values were more notable for the RNA and showed only average results for the ASFV-DNA releasing. Although no significant differences for all the tested releasing methods can be ascertained, the Chelex^®^ 100 Resin methods (M6-E2 and M7-E2) provided excellent results for viral DNA and RNA releasing. The good results were independent of the used amplification method PCR with or without additional extraction. This result was consistent with similar investigations using Chelex^®^ 100 Resin for the development of a direct qRT-PCR for SARS-CoV-2 [19].

Our data support that direct PCR amplification could be suitable for ASFV detection by using FTA cards, which is probably based on the stability of the viral DNA genome of ASFV and the robustness of the used PCR master mix. The direct PCR is a fast, sensitive, and cost-effective method for the detection of ASFV. In contrast, the released viral RNA is less stable than DNA [12] and needs an additional extraction procedure for the inhibition-free RNA isolation.

All used FTA cards could store and release the viral genome of both viruses. After releasing, extraction, and amplification, all cards showed more or less comparable Ct values. The most appropriate cards for ASFV-DNA isolation were the GenSaver 2.0, which was followed by COPAN nucleic acid, GenSaver, and the FTA classic cards, while GenSaver 2.0 and FTA classic cards were more suitable for the IAV detection. Similar results could be ascertained by using FTA classic cards for the detection of both RNA and DNA [12,20]. The feature of using indicating FTA cards could be also demonstrated by the study of other groups [21,22].

It must be noted that the loss on analytical sensitivity by using FTA cards is higher for RNA than DNA approaches. Compared with the liquid original sample material, nearly 5 to 7 Ct values will be lost for the ASFV-DNA amplification via direct PCR after FTA storage and release. In contrast, the best IAV releasing and extraction procedures lost approximately 9 to 11 Ct values compared to the original material. One FTA card spot with a diameter of 2.5 cm and approximately 4.9 cm^2^ will be spiked with 120 µL of the sample material. Only a small part of the spiked card will be used for further analyses. In our study, we used 3 punches with a diameter of 0.3 cm (each 0.07 cm^2^) reflecting approximately 0.21 cm^2^. Thus, the releasing of the nucleic acid from the FTA card represents less than 5% of the original sample. Based on this reflection and the knowledge that the testing of less than 5% of the original sample volume, which would result in an estimated Ct value increase of ≈4.3, it can be concluded that substantial amounts of DNA will be released from the FTA card. The releasing of RNA is markedly decreased compared to the releasing of DNA (at least by a factor of 10), and the reduced recovery will be most likely caused by clogging of the single-stranded RNA in the fiber matrix of the cards based on the complex secondary and tertiary structure. The partial destroying of the viral RNA by the chemicals on the membrane are not likely, because here, substantial differences between the cards and the recovery over the time would be expected.

Based on the data in our study, different nucleic acid releasing methods and commercial FTA cards were successfully applied for the detection of ASFV and IAV. Thus, the FTA card was considered as a reliable diagnostic tool for the storing and extracting of DNA and RNA viruses. This could be applicable in different labs based on the time and costs saving, while in the field, it also fits the purpose of molecular epidemiology research due to its easy transportation and sampling. The data presented here are based on standardized, experimentally generated samples and are therefore not necessarily the same as samples from the field. Nevertheless, it can be assumed that even if the sample quality is poor, the results of the methods used here should be very comparable. The prerequisite for this is that the FTA cards are used according to the manufacturer’s specifications and are not overloaded.

## 4. Materials and Methods

### 4.1. Sample Collection/Viruses

For the generation of the 10-fold dilution series of ASFV positive sample material, an EDTA blood specimen collected from a domestic pig inoculated with ASFV strain Belgium/2018 was used. The trial was performed for strain characterization and reference material acquisition (approved by the competent authority, Landesamt für Landwirtschaft, Lebensmittelsicherheit und Fischerei (LALLF) Mecklenburg-Vorpommern, Rostock, Germany, under reference number 7221.3-2.011/19). For the 10-fold IAV dilution series, allantoid fluid from eggs infected with *Influenza A virus* (A/Mallard/Germany/2009/H5/N3) was applied. Dilution series have been performed from 10 ^−1^ to 10 ^−4^ for both viruses. Based on the different FTA card types and different DNA/RNA releasing methods, we tested 28 EDTA blood samples for the ASFV detection and 28 allantoid fluid samples for the IAV detection.

### 4.2. FTA Cards

The following FTA cards were used in the study:

1. FTA classic card (GE Healthcare Life Science-Whatman, Buckinghamshire, UK), an FTA card that is suitable for the isolation, purification, and storage of nucleic acids.
2. Indicating FTA Elute micro card (GE Healthcare Life Science-Whatman, Buckinghamshire, UK), an FTA card that is designed to simplify the handling, processing, and isolation of nucleic acids.
3. GenSaver (Ahlstrom-Munksjö Germany GmbH, Bärenstein, Germany) is a collection card that is suitable for direct amplification from a paper punch/disc, thus eliminating the extraction step.
4. GenSaver 2.0 (Ahlstrom-Munksjö Germany GmbH, Bärenstein, Germany) is a collection card that is designed for the collection, transport, and storage at ambient temperature of DNA from biological fluids. The fiber-based material of these cards is made of pure absorbent fibers impregnated with a property chemical formulation intended to prevent environmentally-induced degradation of long-term ambient preservation of DNA.
5. Human ID Bloodstain card (GE Healthcare Life Science-Whatman, Buckinghamshire, UK) is a card that is made from absorbent filter paper and designed for the collection and transport of blood and bodily fluids. It is appropriate for short-term handling of specimens.
6. Copan nucleic card (Copan Flock Technologies Srl, Brescia, Italy) is designed to collect, transport and store human DNA from buccal cells, saliva, blood, etc. The lysis treatment on the nucleic card allows a direct PCR short-tandem repeats (STR) analysis on a small punch of the card, without the need for the extraction step.
7. Nucleocard is a blood sample storage card (Macherey-Nagel, Düren, Germany) and FTA card that contains an impregnated specialized filter paper and designed for blood storage for subsequent DNA extraction.

First, 120 µL of each sample were spotted on each FTA card type and left for 48 h to be dried. After spotting and drying, the cards were stored at −20 °C to reduce any damage of nucleic acid under ambient conditions [12]. All steps for the nucleic acid releasing from the cards were conducted at room temperature.

### 4.3. Nucleic Acid-Releasing Methods

By using seven different nucleic acid-releasing methods, eleven eluates were created. Four extra eluates (eluate 1) were created from methods 2, 3, 5, and 6. The cause of generating eluate 1 was trying to reduce the time of releasing process and to show if there are variations between eluate 1 and 2. The selected releasing methods were based on the publications of the supplier, published protocols, and our own experiences. In general, 3 punches of 3 mm size were punched out from each FTA card with a Rayher punch pliers 3.0 mm (Rayher Hobby GmbH, Laupheim, Germany) and then transferred into a 2 mL Eppendorf tube. All eleven supernatants were tested directly in the ASFV and AIV real-time PCR. In addition, 100 µL of the releasing supernatant were extracted with the NucleoMagVet kit (Machery-Nagel, Düren, Germany) on the KingFisher Flex extraction system (ThermoFisher, Darmstadt, Germany), followed by qPCR and RT-qPCR. All card types were processed using the different following procedures:

1. Method 1 (M1), Nucleic acids (DNA and RNA) isolation using FTA purification reagent (GE Healthcare Life Science-Whatman, Buckinghamshire, UK) and proteinase K (Indical Bioscience, Leipzig, Germany): Here, 200 µL of FTA purification reagent and 20 µL of proteinase K were added to the FTA card punches. Afterwards, they were vortexed for 15 s, incubated at 1400 rpm in a thermal shaker at 56 °C for 60 min, and then left to be cooled at room temperature for 5 min. After centrifugation at 7000× *g* for 30 s, the supernatant was transferred in a new reaction tube. The output from FTA card pieces was used as a PCR template for the direct qPCR amplification and as an input sample material for the further nucleic acid extraction.
2. Method 2 (M2-E1), Nucleic acid isolation using FTA Elute buffer (Qiagen, Hilden, Germany), Tris EDTA (TE buffer) (Sigma-Aldrich, St. Louis, MO, USA) and Proteinase K (Indical Bioscience): 500 µL of 1× TE buffer (pH 8.0) were added to the FTA punches, vortexed for 5 s, and then, the supernatant was taken and stored as eluate 1 to be used for the further extraction and direct PCR amplification.
3. Method 2 (M2-E2), Following the last step from M2-E1, FTA card punches were washed 2 times with TE buffer, and afterwards, the supernatants were discarded. Then, 400 µL of FTA Elute buffer (Qiagen GmbH, Hilden, Germany) and 14 µL of Proteinase K were added, which was followed by incubation at 1000 rpm, 60 °C for 25 min, and then incubation at 1000 rpm, 90 °C for 5 min in a thermal shaker. After centrifugation at 7000× *g* for 30 s, the supernatant was transferred to a new Eppendorf tube and stored as eluate 2 and used for both nucleic acid extraction and for the direct PCR amplification.
4. Method 3 (M3-E1), Nucleic acid isolation using Tissue Lysis Reagent (TLR) (Indical Bioscience) and TE buffer (Sigma-Aldrich): TLR buffer has been successfully used for the direct RT-qPCR of Bovine viral diarrhea virus genome from ear notch samples [23]. The punches were taken as described before. First, 500 µL of TE buffer were added, vortexed for 5 s, and then, the supernatant was taken and stored as eluate 1 to be used for further extraction and direct PCR amplification.
5. Method 3 (M3-E2), Subsequently, FTA card punches were washed 2 times with TE buffer, and afterwards, the supernatants were discarded. Then, 400 µL of TLR were added, followed by incubation at 1000 rpm, 60 °C for 25 min, and then incubation at 1000 rpm, 90 °C for 5 min in a thermal shaker. After centrifugation at 7000× *g* for 30 s, the supernatant was transferred to a new Eppendorf tube and used as eluate 2 for both nucleic acid extraction and direct PCR amplification.
6. Method 4 (M4), Nucleic acid isolation using TE buffer (pH 8.0, Sigma-Aldrich): 500 µL of TE buffer were added to the FTA punches and then incubated at 1000 rpm, 26 °C for 30 min in a thermal shaker, which was followed by centrifugation at 7000× *g* for 30 s. The supernatant was transferred to a new Eppendorf tube and used for both nucleic acid extraction and for the direct PCR amplification. TE has been successfully used for nucleic acid releasing [12,16,17].
7. Method 5 (M5), Nucleic acid isolation using complete lysis-M reagent (Roche Diagnostics GmbH, Mannheim, Germany): 400 µL of M-lysis reagent were added to the three punches and then incubated at 1000 rpm, 26 °C for 30 min in a thermal shaker, which was followed by centrifugation at 7000× *g* for 30 s. The supernatant was transferred to a new Eppendorf tube and used for both nucleic acid extraction and for the direct PCR amplification. This buffer was still successfully used for the viral RNA releasing from FTA cards [24].
8. Method 6 (M6-E1), Nucleic acid isolation using Chelex^®^ 100 Resin (Bio-Rad Laboratories, Inc., Hercules, CA, USA) and TE buffer (pH8.0, Sigma-Aldrich): 500 µL of TE buffer were added to the punches followed by the incubation at 1000 rpm, 26 °C for 30 min in a thermal shaker, followed by centrifugation at 7000× *g* for 30 s. The supernatant was transferred to a new Eppendorf tube and stored as eluate 1 [19].
9. Method 6 (M6-E2): First, 500 µL of a 5% *w/v* suspension of Chelex^®^ 100 Resin in sterile water were added to the punches, which was followed by incubation at 1000 rpm at 60 °C for 25 min and at 90 °C for 5 min in a thermal shaker. After centrifugation at 20,000× *g* for 3 min, the supernatant was transferred to new Eppendorf tube and then used as eluate 2 for both the nucleic acid extraction and for the direct PCR amplification.
10. Method 7 (M7-E1), Nucleic acid isolation using Chelex^®^ 100 Resin (Bio-Rad Laboratories, Inc, Hercules, CA, USA) and TED10, which consisted of TE buffer including 10% of dimethylsulfoxid (Carl Roth GmbH, Karlsruhe, Germany): This TED10 solution has been used successfully for the effective viral RNA releasing and direct amplification of SARS-CoV-2 [19]. First, 500 µL of TED10 (90% TE buffer + 10% DMSO) were added to the 3 taken punches from each card and then incubated at 1000 rpm, 26 °C for 15 min in a thermal shaker, which was followed by centrifugation at 7000× *g* for 30 s. The supernatant was transferred to a new Eppendorf tube and stored as eluate 1.
11. Method 7 (M7-E2), 500 µL of 5% *w/v* suspension of Chelex^®^ 100 Resin in sterile water were added, which was followed by incubation at 60 °C for 25 min and then at 90 °C for 5 min in a thermal shaker at 1000 rpm. After centrifugation at 20,000× *g* for 3 min, the supernatant was transferred to a new Eppendorf tube and was used as eluate 2 for both nucleic acid extraction and for the direct PCR amplification.

### 4.4. DNA/RNA Extraction

For the magnetic bead-based extraction, the NucleoMagVet Kit (Macherey-Nagel, Düren, Germany) was conducted on the KingFisher Flex System (Thermo Fisher Scientific, Darmstadt, Germany). Briefly, 100 µL of sample volume were added to 100 µL VL1 lysis buffer and processed according to the instructions of the manufacturer. For internal control, 10 µL IC2-DNA/RNA [25] were mixed with 350 µL VEB binding buffer per sample and added to the sample–lysis buffer mixture. After three washing steps, the extracted nucleic acid was eluted in 100 µL elution buffer. The extraction protocol on the KingFisher Flex System needs approximately 20 min for up to 96 samples. Details of the KingFisher run protocol can be provided on request. The extracted template nucleic acids were stored at −20 °C until use.

### 4.5. Real-Time PCR

*African swine fever virus* (ASFV) detection: The ASFV qPCR assay described by Haines et al. [26] was modified by using a lab-specific amplification mix and the integration of a lab-specific internal control system [18]. Very concretely, for the amplification, the PerfeCTa^®^ qPCR ToughMix^®^ Kit from Quanta BioSciences (Gaithersburg, MD, USA) was applied. A FAM-labeled ASFV primer–probe mixtures consisting of 800 nM primer ASFV-p72IVI-F (5′-GAT GAT GAT TAC CTT YGC TTT GAA-3′), 800 nM primer ASFV-p72IVI-R (5′-TCT CTT GCT CTR GAT ACR TTA ATA TGA-3′), and 200 nM probe ASFV-p72IVI-FAM (5′-FAM-CCA CGG GAG GAA TAC CAA CCC AGT G-BHQ1-3′) in 0.1 × TE buffer (pH 8.0) was realized. For the control of extraction and qPCR, a heterologous control system, published by Hoffmann et al. (2006) [25], was integrated. Here, a HEX-labeled primer-probe-mixture consisting of 200 nM primer EGFP1-F (5′-GAC CAC TAC CAG CAG AAC AC-3′), 200 nM primer EGFP2-R (5′-GAA CTC CAG CAG GAC CAT G-3′), and 200 nM EGFP-probe 1 (5′-HEX-AGC ACC CAG TCC GCC CTG AGC A-BHQ1-3′) in 0.1 × TE buffer (pH 8.0) was prepared. The 12.5 µL total reaction mix was established by 1.75 µL of RNase free water, 6.25 µL of 2 × PerfeCTa qPCR ToughMix, 1.0 µL of ASFV primer probe mix (ASFV-P72-IVI-Mix-FAM), 1.0 µL of the internal control primer probe mix (EGFP-Mix1-HEX), and 2.5 µL DNA template. The following thermo-profile was used for the amplification: 3 min at 95 °C, 45 cycles at 95 °C for 15 s, 60 °C for 20 s, and 75 °C for 20 s. The fluorescence data in the FAM and HEX channel were collected during the annealing step, and the total run time on the CFX96 real-time detection system (Bio-Rad, Hercules, CA, USA) could be ascertained with 1 h and 16 min. For data analyses, the Bio-Rad Maestro software (Version: 4. 1.2433. 1219) was used.

*Influenza A virus* (IAV) detection: Real-time RT-qPCR was performed using the AgPathID One-Step RT-qPCR kit (Applied Biosystems, Foster City, CA, USA). The composition of a single reaction of 12.5 μL was as follows: 1.25 μL of RNase-free water, 6.25 μL of 2 × RT-PCR buffer, 0.5 μL of RT-PCR Enzyme Mix, 1 μL of primer–probe mix for the internal control (EGFP-Mix1-HEX) and 1 μL of IAV specific primer–probe mix. Finally, 2.5 μL of RNA template was added. Cycling conditions were 50 °C for 30′, 94 °C for 2′ min, and 45 cycles of 15″ at 94 °C and 30″ at 56 °C and 30″ at 68 °C. Fluorescence was measured during the 56 °C annealing/extension step. Nuclease-free water served as negative control in all experiments. Briefly, the FAM-labeled IAV-NP2 primer–probe mixtures consisted of 600 nM primer NP-1448-F (5′-GGG AGT CTT CGA GCT CTC-3′), 600 nM primer NP-1543-R (5′-GCA TTG TCT CCG AAG AAA TAA GA-3′), and 200 nM probe IAV-NP-1473-FAM (5′-FAM- AAG GCA VCG ARC CCG ATC GTGC-TAMRA-3′), in 0.1 × TE buffer (pH 8.0). For the internal control amplification, the EGFP-Mix1-HEX, as described above, was used.

Dilution series of an ASFV DNA (ASFV Estonia 2014) and IAV RNA (IAV H5N3) standard were applied in each PCR run to confirm the sensitivity and reproducibility of the performed analyses (Supplementary Tables S1 and S2).

### 4.6. Direct qPCR Amplification

The supernatants of the card’s punches including the released nucleic acid were added directly to the master mix for PCR analysis without prior processing. For the detection of ASFV and IAV, the same PCR assays and kits were used as mentioned above. For internal process control, the internal control assay was changed. Here, 1.0 µL internal control primer–probe mix (Beta-Actin-Mix2-HEX) was used instead of the EGFP–Mix1–HEX described before. Briefly, HEX-labeled Beta-Actin-Mix2 primer–probe mixtures consisting of 600 nM primer ACT-1005-F (5′-CAGCACAATGAAGATCAAGATCATC-3′), 600 nM primer ACT-1135-R (5′-CGGACTCATCGTACTCCTGCTT- 3′), and 200 nM probe ACT-1081-HEX (5′-HEX-TCGCTGTCCACCTTCCAGCAGATGT-BHQ1-3′), in 0.1 × TE buffer (pH 8.0).

### 4.7. Statistical Analysis

Initial data recording and analyses comparison of mean values and transformation of values (comparison of the mean Ct values for each serial dilution based on the 11 different releasing methods, comparison of the mean Ct values for each serial dilution based on the 7 different FTA cards and estimating the delta Ct values between the released output of the FTA card and the extracted original sample based on the different releasing methods and different FTA cards) were done using Microsoft Excel 2010 (Microsoft Germany GmbH, Munich, Germany). GraphPad Prism 8 (GraphPad software Inc., San Diego, CA, USA) was used for further statistical analyses and graph creation. Statistically significant differences were investigated by the statistical test (unpaired multiple *t*-tests) to test the significance of the results (comparison between direct PCR and extraction and then PCR among the different assays and FTA cards; see Supplementary Table S4). Statistical significance would be defined as *p* < 0.05 with an asterisk (*).

## 5. Conclusions

The use of FTA cards seems to be a feasible and easy way for the storage and transport of biological samples for molecular testing. Although all tested FTA cards and releasing methods tested in this study can be applied successfully for the recovery of ASFV-DNA and IAV-RNA, slight differences exist in the analytical sensitivity of the used cards and releasing methods. Interestingly, the direct PCR of ASFV genome delivered moderately (not statistically significant) lower Ct values than samples that underwent a separate extraction. Given the lack of improvement with a second step, the more efficient lab process of direct PCR is sufficient, and additional steps not warranted for general use. In contrast, only a reduced amount of IAV RNA could be amplified from the FTA card by direct RT-qPCR and an increased sensitivity (although not statistically significant) was noted after performing an additional nucleic acid extraction step. For this reason, depending on the downstream analyses, a secondary extraction step may be required and recommended for the RNA detection. Nevertheless, the molecular analyses of strong positive samples via FTA cards can be a helpful option in the diagnostics of pathogens circulating worldwide.

## Figures and Tables

**Table 1:** Qualitative PCR results for 11 DNA/RNA releasing methods using seven different FTA cards and four dilution steps of ASFV DNA and IAV RNA (the positive cards based on each isolation method and dilution series are presented, number of PCR positives/number of FTA brands used).

Method Type	ASFV-qPCR Amplification										IAV-RT-qPCR Amplification									
	Direct qPCR					qPCR after DNA Extraction					Direct RT-qPCR					RT-qPCR after RNA Extraction				
	Dilution Series					Dilution Series					Dilution Series					Dilution Series				
	10^−1^	10^−2^	10^−3^	10^−4^	Sum	10^−1^	10^−2^	10^−3^	10^−4^	Sum	10^−1^	10^−2^	10^−3^	10^−4^	Sum	10^−1^	10^−2^	10^−3^	10^−4^	Sum
M1	7/7	7/7	7/7	2/7	23	7/7	7/7	7/7	1/7	22	7/7	7/7	0/7	0/7	14	7/7	7/7	7/7	1/7	22
M2-E1	7/7	7/7	7/7	3/7	24	7/7	7/7	7/7	1/7	22	7/7	7/7	0/7	0/7	14	7/7	7/7	5/7	1/7	20
M2-E2	7/7	7/7	7/7	7/7	28	7/7	7/7	7/7	3/7	24	7/7	7/7	3/7	0/7	17	7/7	7/7	7/7	2/7	23
M3-E1	7/7	7/7	7/7	0/7	21	7/7	7/7	5/7	0/7	19	7/7	3/7	0/7	0/7	10	7/7	7/7	4/7	0/7	18
M3-E2	7/7	7/7	7/7	4/7	25	7/7	7/7	7/7	4/7	25	7/7	7/7	3/7	0/7	17	7/7	7/7	7/7	1/7	22
M4	7/7	7/7	7/7	1/7	22	7/7	7/7	5/7	1/7	20	7/7	7/7	0/7	0/7	14	7/7	7/7	7/7	2/7	23
M5	7/7	7/7	7/7	0/7	21	7/7	7/7	5/7	0/7	19	7/7	7/7	0/7	0/7	14	7/7	7/7	7/7	1/7	22
M6-E1	7/7	7/7	6/7	0/7	20	7/7	7/7	3/7	0/7	17	7/7	5/7	0/7	0/7	12	7/7	7/7	5/7	0/7	19
M6-E2	7/7	7/7	7/7	4/7	25	7/7	7/7	7/7	5/7	26	7/7	7/7	7/7	2/7	23	7/7	7/7	7/7	4/7	25
M7-E1	7/7	7/7	5/7	1/7	20	7/7	7/7	5/7	0/7	19	7/7	6/7	0/7	0/7	13	7/7	7/7	5/7	0/7	19
M7-E2	7/7	7/7	7/7	5/7	26	7/7	7/7	7/7	4/7	25	7/7	7/7	7/7	2/7	23	7/7	7/7	7/7	4/7	25

ASFV (*African swine fever virus*), IAV (*Influenza A virus*), (M1) Method 1 (FTA Purification Reagent + Proteinase K), (M2-E1) Method 2–Eluate 1 (TE buffer + Proteinase K + FTA Elute buffer), (M2-E2) Method 2–Eluate 2, (M3-E1) Method 3–Eluate 1 (TE buffer + TLR buffer), (M3-E2) Method 3–Eluate 2, (M4) Method 4 (TE buffer), (M5) Method 5 (M-lysis Reagent), (M6-E1) Method 6–Eluate 1 (TE buffer + Chelex^®^ 100 Resin), (M6-E2) Method 6–Eluate2, (M7-E1) Method 7–Eluate 1 (TED10 + Chelex^®^ 100 Resin), (M7-E2) Method 7–Eluate 2.

**Table 2:** Qualitative data analysis of the values of the different DNA/RNA eleven releasing methods based on the seven tested FTA cards (the positive methods based on each card type and dilution series are shown, number of PCR positives/number of releasing methods used).

Card Type	ASFV-qPCR Amplification										IAV-RT-qPCR Amplification									
	Direct qPCR					qPCR after DNA Extraction					Direct RT-qPCR					RT-qPCR after RNA Extraction				
	Dilution Series					Dilution Series					Dilution Series					Dilution Series				
	10^−1^	10^−2^	10^−3^	10^−4^	Sum	10^−1^	10^−2^	10^−3^	10^−4^	Sum	10^−1^	10^−2^	10^−3^	10^−4^	Sum	10^−1^	10^−2^	10^−3^	10^−4^	Sum
1	11/11	11/11	11/11	4/11	37	11/11	11/11	10/11	0/11	32	11/11	11/11	2/11	0/11	24	11/11	11/11	11/11	5/11	38
2	11/11	11/11	11/11	2/11	35	11/11	11/11	6/11	1/11	29	11/11	11/11	2/11	0/11	24	11/11	11/11	11/11	3/11	36
3	11/11	11/11	10/11	3/11	35	11/11	11/11	9/11	3/11	34	11/11	11/11	4/11	2/11	28	11/11	11/11	10/11	2/11	34
4	11/11	11/11	11/11	7/11	40	11/11	11/11	11/11	5/11	38	11/11	8/11	4/11	0/11	23	11/11	11/11	11/11	5/11	38
5	11/11	11/11	9/11	4/11	35	11/11	11/11	8/11	5/11	35	11/11	10/11	4/11	2/11	27	11/11	11/11	11/11	0/11	33
6	11/11	11/11	11/11	4/11	37	11/11	11/11	11/11	1/11	34	11/11	9/11	2/11	4/11	26	11/11	11/11	7/11	0/11	29
7	11/11	11/11	11/11	3/11	36	11/11	11/11	10/11	4/11	36	11/11	10/11	2/11	3/11	26	11/11	11/11	7/11	1/11	30

1 FTA classic card, 2 Indicating FTA Elute card, 3 GenSaver, 4 GenSaver 2.0, 5 Human ID Bloodstain card, 6 COPAN Nucleic card, 7 Nucleo card/Blood sample storage card.

## Data Availability

The data set used and/or analyzed during the current study are available from the corresponding author on reasonable request.

## Supplementary Materials

The following are available online at https://www.mdpi.com/article/10.3390/ijms222312915/s1.

## References

[1] Rahikainen A.-L., Palo J.U., de Leeuw W., Budowle B., Sajantila A. (2016). DNA quality and quantity from up to 16 years old post-mortem blood stored on FTA cards. Forensic Sci. Int..

[2] Pezzoli N., Silvy M., Woronko A., Le Treut T., Levy-Mozziconacci A., Reviron D., Gabert J., Picard C. (2007). Quantification of mixed chimerism by real time PCR on whole blood-impregnated FTA cards. Leuk. Res..

[3] Liang X., Chigerwe M., Hietala S.K., Crossley B.M. (2014). Evaluation of Fast Technology Analysis (FTA) Cards as an improved method for specimen collection and shipment targeting viruses associated with Bovine Respiratory Disease Complex. J. Virol. Methods.

[4] Abdelwhab E., Lüschow D., Harder T., Hafez H.M. (2011). The use of FTA^®^ filter papers for diagnosis of avian influenza virus. J. Virol. Methods.

[5] Perozo F., Villegas P., Estevez C., Alvarado I., Purvis L.B. (2006). Use of FTA filter paper for the molecular detection of Newcastle disease virus. Avian Pathol..

[6] Inoue R., Tsukahara T., Sunaba C., Itoh M., Ushida K. (2007). Simple and rapid detection of the porcine reproductive and respiratory syndrome virus from pig whole blood using filter paper. J. Virol. Methods.

[7] Maw M.T., Yamaguchi T., Kasanga C.J., Terasaki K., Fukushi H. (2006). A practical tissue sampling method using ordinary paper for molecular detection of infectious bursal disease virus RNA by RT-PCR. Avian Dis..

[8] Muthukrishnan M., Singanallur N.B., Ralla K., Villuppanoor S.A. (2008). Evaluation of FTA cards as a laboratory and field sampling device for the detection of foot-and-mouth disease virus and serotyping by RT-PCR and real-time RT-PCR. J. Virol. Methods.

[9] Picard-Meyer E., Barrat J., Cliquet F. (2007). Use of filter paper (FTA) technology for sampling, recovery and molecular characterisation of rabies viruses. J. Virol. Methods.

[10] Braae U.C., Johansen M.V., Ngowi H.A., Rasmussen T.B., Nielsen J., Uttenthal Å. (2015). Detection of African swine fever virus DNA in blood samples stored on FTA cards from asymptomatic pigs in Mbeya region, Tanzania. Transbound. Emerg. Dis..

[11] Mason M.G., Botella J.R. (2020). Rapid (30-s), equipment-free purification of nucleic acids using easy-to-make dipsticks. Nat. Protoc..

[12] Sakai T., Ishii A., Segawa T., Takagi Y., Kobayashi Y., Itou T. (2015). Establishing conditions for the storage and elution of rabies virus RNA using FTA^®^ cards. J. Vet. Med. Sci..

[13] Rodiño J.M., Aguilar Y.A., Rueda Z.V., Vélez L.A. (2016). Usefulness of FTA^®^ cards as a Pneumocystis-DNA extraction method in bronchoalveolar lavage samples. Infect. Dis..

[14] Saieg M.A., Geddie W.R., Boerner S., Liu N., Tsao M., Zhang T., Kamel-Reid S., Santos G.D.C. (2012). The use of FTA cards for preserving unfixed cytological material for high-throughput molecular analysis. Cancer Cytopathol..

[15] da Cunha Santos G. (2018). FTA Cards for Preservation of Nucleic Acids for Molecular Assays: A Review on the Use of Cytologic/Tissue Samples. Arch. Pathol. Lab. Med..

[16] Manswr B., Ball C., Forrester A., Chantrey J., Ganapathy K. (2018). Evaluation of full S1 gene sequencing of classical and variant infectious bronchitis viruses extracted from allantoic fluid and FTA cards. Avian. Pathol..

[17] Veiga I.B., Mühldorfer K., Hafez H.M., Lüschow D. (2020). Whatman^®^ FTA^®^ Cards Performance for Ornithobacterium rhinotracheale DNA Amplification. Avian Dis..

[18] Elnagar A., Pikalo J., Beer M., Blome S., Hoffmann B. (2021). Swift and Reliable “Easy Lab” Methods for the Sensitive Molecular Detection of African Swine Fever Virus. Int. J. Mol. Sci..

[19] Guan B., Frank K.M., Maldonado J.O., Beach M., Pelayo E., Warner B.M., Hufnagel R.B. (2021). Sensitive extraction-free SARS-CoV-2 RNA virus detection using a chelating resin. iScience.

[20] Johanson H.C., Hyland V., Wicking C., Sturm R.A. (2009). DNA elution from buccal cells stored on Whatman FTA Classic Cards using a modified methanol fixation method. Biotechniques.

[21] Wang D.Y., Chang C.-W., Lagacé R.E., Oldroyd N.J., Hennessy L.K. (2011). Development and validation of the AmpFℓSTR^®^ Identifiler^®^ Direct PCR Amplification Kit: A multiplex assay for the direct amplification of single-source samples. J. Forensic Sci..

[22] Stangegaard M., Børsting C., Ferrero-Miliani L., Frank-Hansen R., Poulsen L., Hansen A.J., Morling N. (2013). Evaluation of four automated protocols for extraction of DNA from FTA cards. J. Lab. Autom..

[23] Wernike K., Beer M. (2019). Diagnostics in the context of an eradication program: Results of the German bovine viral diarrhea proficiency trial. Vet. Microbiol..

[24] Yacouba A., Congo M., Dioma G.K., Somlare H., Coulidiaty D., Ouattara K., Sangare L. (2020). Whatman FTA cards versus plasma specimens for the quantitation of HIV-1 RNA using two real-time PCR assays. Access Microbiol..

[25] Hoffmann B., Depner K., Schirrmeier H., Beer M. (2006). A universal heterologous internal control system for duplex real-time RT-PCR assays used in a detection system for pestiviruses. J. Virol. Methods.

[26] Haines F.J., Hofmann M.A., King D., Drew T., Crooke H.R. (2013). Development and validation of a multiplex, real-time RT PCR assay for the simultaneous detection of classical and African swine fever viruses. PLoS ONE.

